# Treatment of Fistula in Ano by Ligature

**Published:** 1845-12

**Authors:** 


*Treatment of Fistula in Ano by Ligature.* By Mr. Luke.—The advantages of this method over that by the knife are: 1st. The shorter period which usually elapses before the final cure. 2d. The less pain which is felt during treatment. 3. The absence of the dread which the knife generally inspires, and the consequent inducement which it offers to the patient to submit to effective curative treatment; and, lastly the avoidance of all hemorrhage. The treatment is to be conducted in the following manner: An eyed probe, armed with dentist's silk, is introduced through the fistula into the rectum, from whence the silk is withdrawn through the anus, by means of a spring-catch introduced into the rectum upon the finger of the operator. The parts to be divided are then inclosed between the two extremities of the ligature, to which a small fistula-tourniquet is subsequently attached, by passing them through holes provided for the purpose. The requisite amount of tension is maintained by a screw. Care must be taken that the ligature be not so tight as to cause more than slight uneasiness. After the lapse of a few days, ulceration of the enclosed part commences, and the tourniquet becomes loosened, indicating the necessity of the ligature being made tighter.

Nine cases are appended, in which the proceeding was attended with perfect success.—*Lancet.*—*Ibid.*

